# Novel Targets of SARS-CoV-2 Spike Protein in Human Fetal Brain Development Suggest Early Pregnancy Vulnerability

**DOI:** 10.3389/fnins.2020.614680

**Published:** 2021-01-21

**Authors:** Parul Varma, Zane R. Lybrand, Mariah C. Antopia, Jenny Hsieh

**Affiliations:** ^1^Department of Biology, University of Texas at San Antonio, San Antonio, TX, United States; ^2^Brain Health Consortium, University of Texas at San Antonio, San Antonio, TX, United States; ^3^Department of Biology, Texas Woman's University, Denton, TX, United States

**Keywords:** SARS-CoV-2, COVID-19, spike protein, fetal brain, pregnancy, BrainSpan, scRNA seq, vertical transmission

## Abstract

Pregnant women are at greater risk of infection by severe acute respiratory syndrome coronavirus 2 (SARS-CoV-2), because of their altered immunity and strained cardiovascular system. Emerging studies of placenta, embryos, and cerebral organoids suggest that fetal organs including brain could also be vulnerable to coronavirus disease 2019 (COVID-19). Additionally, a case study from Paris has reported transient neurological complications in neonates born to pregnant mothers. However, it remains poorly understood whether the fetal brain expresses cellular components that interact with Spike protein (S) of coronaviruses, which facilitates fusion of virus and host cell membrane and is the primary protein in viral entry. To address this question, we analyzed the expression of known (*ACE2, TMPRSS2, and FURIN*) and novel (*ZDHHC5, GOLGA7, and ATP1A1*) S protein interactors in publicly available fetal brain bulk and single cell RNA sequencing datasets. Bulk RNA sequencing analysis across multiple regions of fetal brain spanning 8 weeks post conception (wpc)−37wpc indicates that two of the known S protein interactors are expressed at low levels with median normalized gene expression values ranging from 0.08 to 0.06 (*ACE2)* and 0.01–0.02 (*TMPRSS*2). However, the third known S protein interactor *FURIN* is highly expressed (11.1–44.09) in fetal brain. Interestingly, all three novel S protein interactors are abundantly expressed throughout fetal brain development with median normalized gene expression values ranging from 20.38–21.60 (*ZDHHC5*), 92.47–68.35 (*GOLGA7*), and 65.45–194.5 (*ATP1A1*). Moreover, the peaks of expression of novel interactors is around 12–26wpc. Using publicly available single cell RNA sequencing datasets, we further show that novel S protein interactors show higher co-expression with neurons than with neural progenitors and astrocytes. These results suggest that even though two of the known S protein interactors are present at low levels in fetal brain, novel S protein interactors are abundantly present and could play a direct or indirect role in SARS-CoV-2 fetal brain pathogenesis, especially during the 2^nd^ and 3^rd^ trimesters of pregnancy.

## Introduction

Recent outbreak of severe acute respiratory syndrome coronavirus 2 (SARS-CoV-2) has infected more than 64 million people worldwide and continues to threaten health and economic well-being of people all around the globe (https://coronavirus.jhu.edu/). Despite fast-tracked intensive research on many aspects of SARS-CoV-2, the impact of infection in pregnant mothers and developing fetuses remains poorly explored. Using single cell transcriptome analysis, some studies have suggested that placenta, early embryos and fetal organs such as heart, liver, and lungs are vulnerable to coronavirus disease 2019 (COVID-19) (Ashary et al., 2020; Li et al., 2020; Weatherbee et al., 2020). Additionally, a recent case study has reported transient neurological complications in neonates born to infected mothers, suggesting that the fetal brain could be vulnerable to COVID-19 (Vivanti et al., 2020). Though currently there is a lack of conclusive evidence of infection in fetal brain, studies have shown that SARS-CoV-2 can infect cerebral organoids, which are an *in vitro* 3D model of human fetal brain development (Jacob et al., 2020; Pellegrini et al., 2020; Ramani et al., 2020; Song et al., 2020; Zhang et al., 2020). However, these studies only highlight the possibility of vertical transmission during pregnancy; it remains unknown whether fetal brain expresses cellular components that could lead to SARS-CoV-2 infection and affect fetal neurodevelopment.

Coronaviruses such as SARS-CoV and Middle East respiratory syndrome (MERS) are known to infect animal and human brain (Yamashita et al., 2005; Li et al., 2016). However, the molecular mechanisms by which coronaviruses infect brain cells are not well-known. SARS-CoV-2, the seventh and most recently recognized member of the family *Coronaviridae* (genus *Betacoronavirus*, subgenus *Sarbecovirus*) produces Spike (S) protein, which has been shown to play a major role in human pathogenesis (Wu et al., 2020). S is a surface glycoprotein that engages angiotensin converting enzyme 2 (ACE2) receptor for entry into the target cells. Infection requires that the receptor binding domain (RBD) S1 binds to the host cell receptor, and that site S2 undergoes proteolytic cleavage by Furin protease and/or transmembrane protease serine 2 (TMPRSS2) (Hoffmann et al., 2020). Most of the tissues in human body express *ACE2*, with the highest expression occurring in lung epithelial cells. In the brain, *ACE2* is expressed only in endothelium and vascular smooth muscle cells, which suggests that infection of neurons requires alternate routes of entry (Hamming et al., 2004) It has been shown that SARS-CoV can use alternate receptors such as CD209L for infection and pathogenesis (Jeffers et al., 2004; Yang et al., 2004), and uses cellular receptor neurophilin-1 to invade olfactory bulb epithelium (Cantuti-Castelvetri et al., 2020).

To unveil drug targets for SARS-CoV-2, a recent proteomic study expressed 26 out of 29 SARS-CoV-2 proteins in HEK293T/17 cells and identified their interacting partners. Out of the 332 identified proteins, ZDHHC5 and GOLGA7 showed high-confidence interaction specifically with the S protein of SARS-CoV-2. ATP1A1 also showed specific interaction with S protein but was slightly below the authors' cutoff value (Gordon et al., 2020). ZDHHC5 belongs to the zinc finger Asp-His-His-Cys (DHCC) family of proteins that catalyze protein palmitoylation. Most ZDHHC5 proteins occur in the endoplasmic reticulum (ER) or Golgi apparatus. However, three ZDHHC enzymes (ZDHHC5, ZDHHC20, and ZDHHC21) primarily localize to the plasma membrane (Ohno et al., 2006).

Among the other novel S interactors, Golgin A7 (GOLGA7), is an acylated Golgi protein that forms a protein acyltransferase complex with ZDHHC5 and localizes to the plasma membrane (Ohta et al., 2003; Ko et al., 2019), while the Na^+^, K^+^-ATPase with A1 alpha subunit isoform (ATP1A1) is an integral transmembrane ion transporter, essential for maintaining the electrochemical gradient of Na^+^ and K^+^ ions in excitable cells. *ATP1A1* has been shown to play a crucial role in coronaviruses (CoV) entry (Burkard et al., 2015). These three novel S protein interactors have the potential to affect the physiology of cells exposed to the SARS-CoV-2 virus.

Fetal brain expression of known (*ACE2, TMPRSS2, FURIN*) and novel S protein interactors (*ZDHHC5, GOLGA7*, and *ATP1A1*) suggests fetal brain invasion and impact of SARS-CoV-2 in infected pregnant mothers. We first analyzed the spatio-temporal expression of the S protein interactors *ACE2, TMPRSS2*, and *FURIN* in a publicly available fetal brain transcriptome dataset downloaded from BrainSpan Atlas of the Developing Human Brain (https://brainspan.org/). In this dataset, *ACE2* and *TMPRSS2* are expressed at low levels during fetal brain development, while *FURIN* is highly expressed. Our analysis of novel S protein interactors revealed that *ZDHHC5, GOLGA7*, and *ATP1A1* are abundantly expressed throughout fetal brain development with peaks of expression during the 2^nd^ and 3^rd^ trimester of pregnancy. Using publicly available fetal brain single cell RNA sequencing (scRNA seq) datasets, we further found that *ZDHHC5, GOLGA7*, and *ATP1A1* are expressed in both neural progenitors and neuronal cells but shows higher co-expression with neurons than in progenitors and astrocytes. Our study is the first comprehensive bioinformatic study exploring the expression of known and newly identified S protein interactors in human fetal brain. Further cellular and molecular analysis of these genes will identify their potential for neuroinvasion of SARS-CoV-2 and disruption of fetal brain development.

## Materials and Methods

### Fetal Brain Bulk RNA Sequencing

We downloaded fetal brain bulk RNA sequencing dataset containing RPKM (reads per kilobase transcripts per million mapped reads) values for all genes from the BrainSpan: Atlas of the Developing Human Brain (https://brainspan.org/). BrainSpan is the outcome of a consortium consisting of the Allen Institute for Brain Science, Yale University, the Zilkha Neurogenetic Institute of the Keck School of Medicine of the University of Southern California, the Athinoula A. Martinos Center at Massachusetts General Hospital/Harvard Medical School and MIT HST/CSAIL, the University of California, Los Angeles and the University of Texas Southwestern Medical Center. The gene expression data has been generated using postmortem human brain specimens from 8wpc to 40 years of age. In total, data consists of 31 developmental stages at various wpc, months(mos), and years(yrs). The stages in the dataset are 8wpc, 9wpc, 12wpc, 13wpc, 16wpc, 17wpc, 19wpc, 21wpc, 24wpc, 25wpc, 26wpc, 35wpc, 37wpc, 4mos, 10mos, 1yrs, 2yrs, 3yrs, 4yrs, 8yrs, 11yrs, 13yrs, 15yrs, 18yrs, 19yrs, 21yrs, 23yrs, 30yrs, 36yrs, 37yrs, and 40yrs and include ~26 microdissected brain structures. Since we were only interested in fetal brain expression pattern, we complied the gene expression data for known and novel S protein interactors from 8 to 37wpc and analyzed their expression across various brain regions. However, gene expression data for all the microdissected brain regions was not available for every time point. For example, brain specimen at 8wpc contained expression data for 16 unique microdissected structures but specimen at 35wpc contained data for only two microdissected regions. Therefore, in our analysis, we treated each microdissected brain region for a particular time point as a unique independent sample (*N* = 1), performed secondary qualitative analysis and refrained from doing any statistical quantification between independent samples. However, we normalized the gene expression values in each independent sample by converting RPKM values to TPM (transcripts per million); briefly, the RPKM value for each gene in a sample at a particular time point was converted to TPM using the formula $\frac{RPKM\left(gene\right)}{\sum RPKM\left(all\;genes\;in\;the\;sample\right)}X\;10^{6}\;$(Wagner et al., 2012). We used Graphpad Prism to plot temporal expression pattern of genes of interest and used descriptive statistics to generate and compare median values of expression at each time point. As each median value at a particular time point only provided an estimate of the distribution of gene expression across available brain regions (*N* = 1) at that time point, we did not perform any further quantitative statistical analysis. Heatmaps for spatial expression of genes were made using heatmap function in R where normalized expression values were scaled to column. Heatmaps were not normalized to each other. For simplicity, if there were more than two values of expression for a region of interest at the corresponding timepoint, we averaged the expression values and plotted the average on heatmaps. We retained information about the sex of the fetal donor wherever possible but performed no sex-specific analysis.

### Fetal Brain scRNA Sequencing

Fetal brain scRNA seq datasets were downloaded from publicly available single-cell datasets maintained by the Hemberg lab at Wellcome Trust Sanger Institute (https://hemberg-lab.github.io/scRNA.seq.datasets/human/brain/). The single-cell expression set (SCEset) class of object for single-cell datasets was downloaded from Camp et al. (2015) and Darmanis et al. (2015). Seurat R package was used for all further analysis of single cell gene expression data (Butler et al., 2018; Stuart et al., 2019). In brief, SCEset was first converted to Seurat object using interoperability package of Seurat. While gene expression dataset from Camp et al. (2015) contained data for fetal brain cells at 12–13wpc, Darmanis et al. (2015) contained data for fetal brain cells at 16–18wpc. However, both the datasets contained expression data for other non-fetal non-brain cells as well and therefore, we extracted expression matrix of only fetal brain cells from the main dataset for all downstream analysis. Default parameters in Seurat R package were used for normalization, scaling, and dimensionality reduction of the single cell expression data. Uniform manifold approximation and projection (UMAP) method in Seurat was used for non-linear dimension reduction and visualization of the datasets. Clusters of cells were identified based on the gene-expression markers. We used the WhichCells function of Seurat to identify the total number of cells expressing the gene(s) of interest in each dataset.

## Results

### S Protein Interactors of SARS-CoV-2 Are Not Highly Expressed During Human Fetal Brain Development

**Figure 1:**
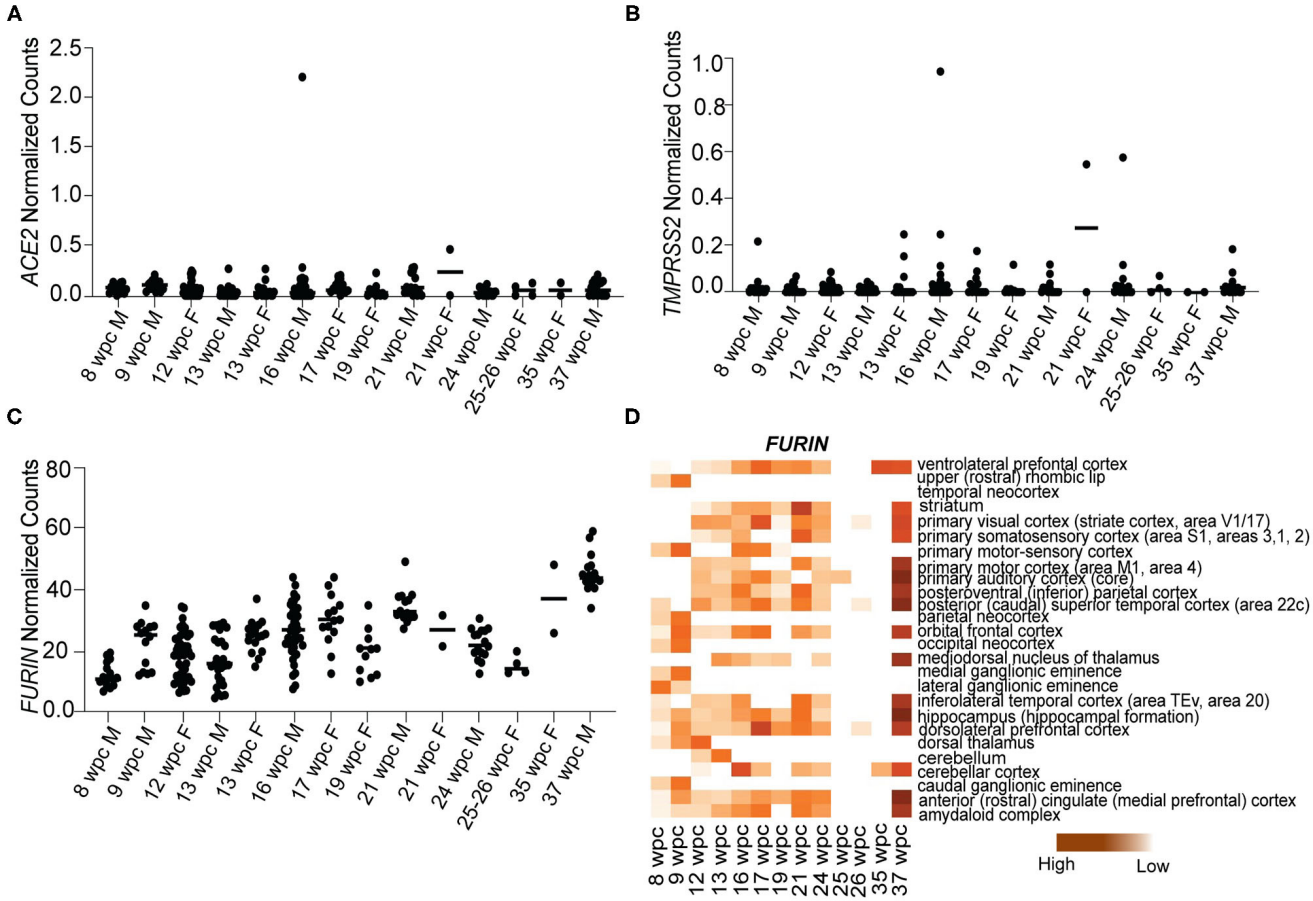
S protein interactors of SARS-CoV-2 are not expressed during human fetal brain development. **(A)** Temporal expression of *ACE2* from 8 to 37wpc during human fetal brain development. **(B)** Temporal expression of *TMPRSS2* from 8 to 37wpc during human fetal brain development. **(C)** Temporal expression of *FURIN* from 8 to 37wpc during human fetal brain development. **(D)** Heatmap representing spatio-temporal expression of *FURIN* across 26 different fetal brain regions ranging from 8 to 37wpc. *FURIN* shows maximum expression at 37wpc. In temporal expression graphs, each value corresponds to a specific region at that time point and horizontal black lines represent median value of gene expression at each time point. The heatmaps are scaled to column. White spaces represent lack of data for regions of interest at the corresponding timepoint.

A number of studies have established ACE2, TMPRSS2, and Furin as the main proteins of SARS-CoV-2 involved in human pathogenesis (Cai et al., 2020; Hoffmann et al., 2020). To study the vulnerability of human fetal brain to SARS-CoV-2 infection, we analyzed the expression of these genes in the fetal brain RNA sequencing dataset downloaded from BrainSpan Atlas of the Developing Human Brain (https://brainspan.org/). This dataset contains gene expression data that has been generated using postmortem human brain specimens from 8wpc to 40 years of age. Since we were only interested in fetal brain expression pattern, we analyzed the gene expression data for genes of interest from 8 to 37wpc across various brain regions. We found that *ACE2* [median normalized counts (NC) ranging from 0.08 to 0.06] and *TMPRSS2* (median range 0.01–0.02 NC) were nominally expressed, while *FURIN* (median range 11.1–44.09 NC) was more expressed throughout fetal brain development (Figures 1A–C). We further analyzed expression of *FURIN* across various brain regions in the RNA transcriptome dataset. Most of the brain regions at 37wpc showed high expression of *FURIN*, with the peak expression value (59.11 NC) occurring in the hippocampus (Figure 1D). Altogether, we observed that, with the exception of *FURIN*, known S protein interactors are not highly expressed during fetal brain development, suggesting that SARS-CoV-2 invades fetal neurons via other interactions.

### Novel S Protein Interactors *ZDHHC5, GOLGA7*, and *ATP1A1* Are Expressed Throughout Human Fetal Brain Development

**Figure 2:**
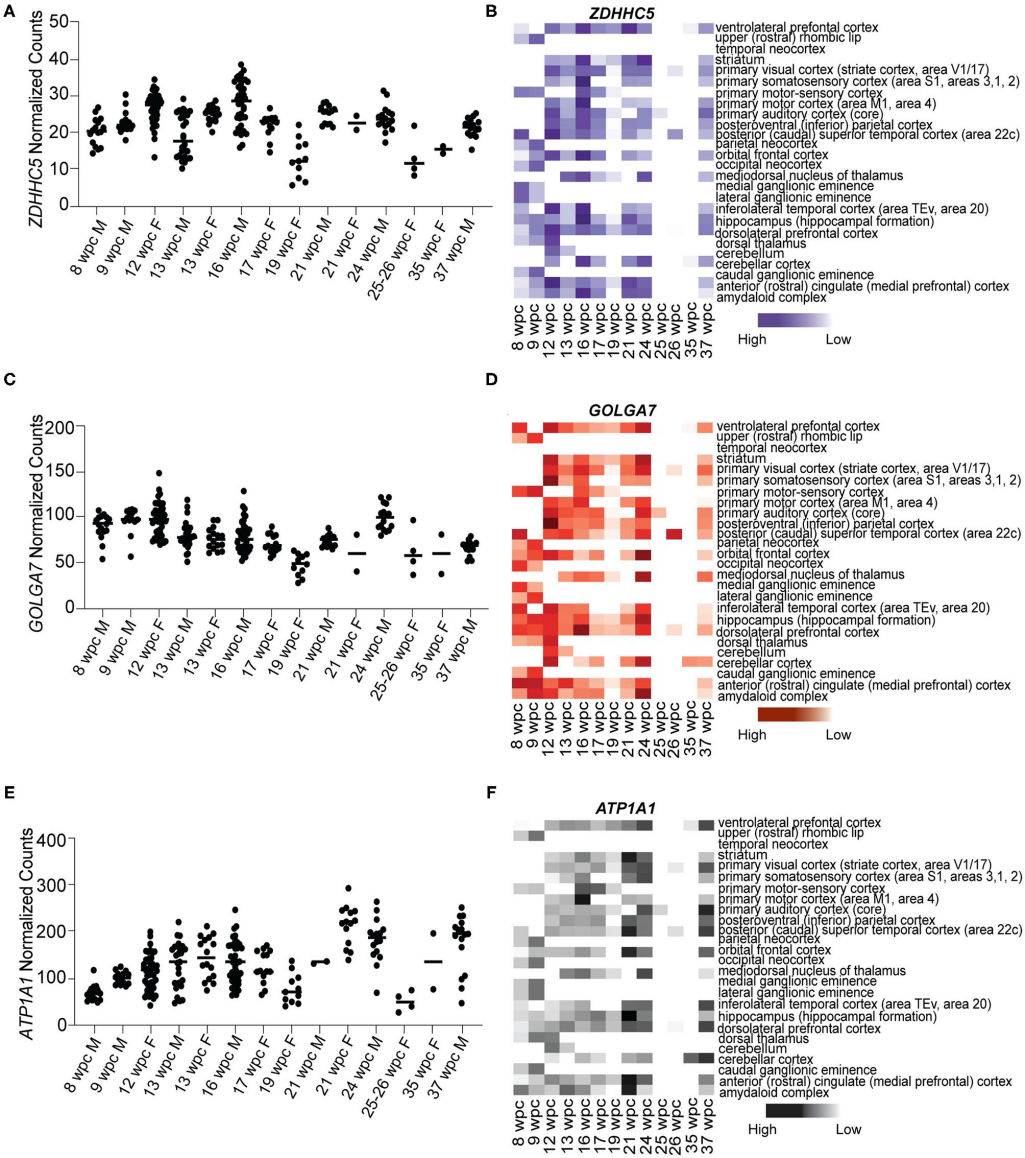
Novel S protein interactors ZDHHC5, GOLGA7, and ATP1A1 are expressed throughout the fetal brain development. **(A)** Temporal expression of *ZDHHC5* from 8 to 37wpc showing three peaks of expression at 12, 16, and 24wpc. **(B)** Heatmap representing spatio-temporal expression of *ZDHHC5* across 26 different fetal brain regions ranging from 8 to 37wpc. **(C)** Temporal expression of *GOLGA7* from 8 to 37wpc. Note peaks of expression at 12, 16, and 24wpc. **(D)** Heatmap representing spatio-temporal expression of *GOLGA7* across 26 different fetal brain regions ranging from 8 to 37wpc. **(E)** Temporal expression of ATP1A1 from 8-37 wpc showing three peaks of expression at 21, 24 and 37 wpc. **(F)** Heatmap representing spatio-temporal expression of ATP1A1 across 26 different fetal brain regions ranging from 8 to 37wpc. In temporal expression graphs, each value corresponds to a specific region at that time point and horizontal black lines represent median value of gene expression at each time point. The heatmaps are scaled to column. White spaces represent lack of data for regions of interest at the corresponding timepoint.

As *ACE2* and *TMPRSS2* are not expressed during fetal brain development, we analyzed the expression of newly identified S protein interactors *ZDHHC5, GOLGA7*, and *ATP1A1* (Gordon et al., 2020). Our analysis revealed that all three genes are expressed abundantly at all stages of development (Figure 2). We found that *ZDHHC5* (median range 20.38–21.60 NC) was moderately expressed during fetal brain development, while *GOLGA7* (median range 92.47–68.35 NC) and *ATP1A1* (median range 65.45–194.5 NC) were abundantly expressed during 8–37wpc (Figures 2A,C,E). Expression of *ZDHHC5* peaked thrice during fetal development; the first time around 12wpc in inferolateral temporal cortex (29.855 NC), the second around 16wpc in primary somatosensory cortex (34.986 NC) and third around 24wpc in striatum (31.356 NC) (Figure 2B). Interestingly, peak expression of *GOLGA7* overlapped with expression of *ZDHHC5*. We found that *GOLGA7* was most abundantly expressed in posteroventral parietal cortex at 12wpc (148.293 NC) followed by dorsolateral prefrontal cortex at 16wpc (128.810 NC) and amygdaloid complex at 24wpc (121.919 NC) (Figure 2D). Lastly, the expression of *ATP1A1* was most abundant in anterior cingulate cortex at 21wpc (292.600 NC) and primary visual cortex at both 24wpc (264.514 NC) and 37wpc (252.05 NC) (Figure 2F). Together these results suggest that although two of the known S protein interactors are expressed at low levels in human fetal brain, newly identified S protein interactors are highly expressed and represent possible targets of SARS-CoV-2 fetal brain infection around the 2^nd^ and 3^rd^ trimester (12–26wpc) of pregnancy.

### Novel S Protein Interactors *ZDHHC5, GOLGA7*, and *ATP1A1* Are Expressed More in Neurons Than Progenitors

**Figure 3:**
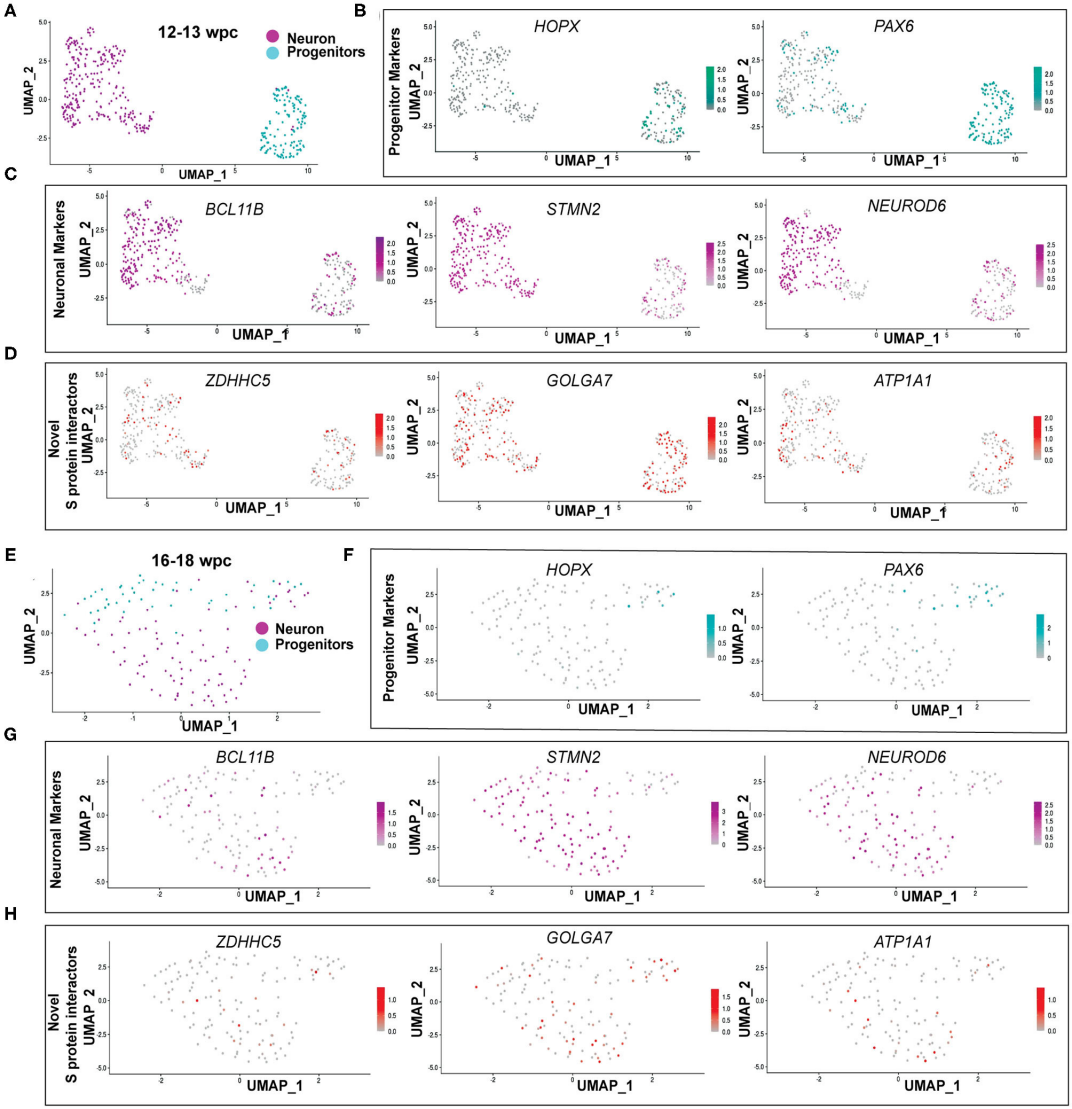
Novel S protein interactors ZDHHC5, GOLGA7, and ATP1A1 are expressed more in neurons than progenitors. **(A)** UMAP of 12**–**13wpc dataset. **(B)** UMAP of 12**–**13wpc dataset with expression levels of outer radial glia (*HOPX*) and progenitor markers (*PAX6*). **(C)** UMAP of 12**–**13wpc dataset with expression levels of neuronal markers (*BCL11B, STMN2*, and *NEUROD6*). **(D)** UMAP of 12**–**13wpc dataset with expression levels of novel S protein interactors *ZDHHC5, GOLGA7*, and *ATP1A1*. **(E)** UMAP of 16–18wpc dataset. **(F)** UMAP of 16–18wpc dataset with expression levels of outer radial glia (*HOPX*) and progenitor markers (*PAX6*). **(G)** UMAP of 16–18wpc dataset with expression levels of neuronal markers (*BCL11B, STMN2*, and *NEUROD6*). **(H)** UMAP of 16–18wpc dataset with expression levels of novel S protein interactors *ZDHHC5, GOLGA7*, and *ATP1A1*.

As *ZDHHC5, GOLGA7*, and *ATP1A1* showed peak expression around 12–24wpc, we further analyzed their expression in publicly available fetal brain scRNA seq datasets at 12–13wpc (Camp et al., 2015) and 16–18wpc (Darmanis et al., 2015). First, we found that all three genes were expressed in neurons and progenitors at both 12–13wpc and 16–18wpc (Figures 3A–H). Second, in order to identify which cell population more strongly expressed these genes, we analyzed the total number of cells in each dataset for co-expression of neuronal and progenitor markers. We found that at both 12–13wpc and 16–18wpc, *ZDHHC5, GOLGA7*, and *ATP1A1* abundantly co-expressed with *DCX* and *NEUROD6* (immature neurons) and *STMN2* (mature neurons) as well as in cells expressing *VIM* (pan radial glial cells), *HOPX* (outer radial glial cells), and *PAX6* (neural progenitor cells). They also showed expression in cortical cells, both in deep layers (*SATB2, CTIP2, TLE4*) and upper layers (*RELN, CUX1, BRN2*). There were very few astrocytes or oligodendrocytes in these datasets and little to no co-expression of *ZDHHC5, GOLGA7*, and *ATP1A1* with astrocyte (*S100B, GFAP*) and oligodendrocyte (*OLIG2*) markers (Table 1). As with the bulk RNA seq dataset, we did not detect expression of *ACE2* and *TMPRSS2* in the fetal brain scRNA seq dataset but found that *FURIN* was expressed in a few neurons and progenitors (Supplementary Figure 1). Overall, these results suggest that though novel S protein interactors are expressed in both neurons and progenitors, there seems to be higher co-expression with neuronal markers, implying that fetal neurons may be more vulnerable to SARS-CoV-2 brain infection around the 2^nd^ and 3^rd^ trimester.

**Table 1:** Co-expression analysis of novel S protein interactors ZDHHC5, GOLGA7, and ATP1A1.

**12–13wpc (Total Cells 380)**				**16–18wpc (Total Cells 135)**			
	**ZDHHC5 (69)**	**GOLGA7 (184)**	**ATP1A1 (78)**		**ZDHHC5 (26)**	**GOLGA7 (65)**	**ATP1A1 (38)**
VIM (213)	36	117	46	VIM (48)	13	22	17
HOPX (50)	12	27	10	HOPX (12)	2	9	5
PAX6 (200)	36	108	41	PAX6 (37)	6	19	9
EOMES (32)	11	15	2	EOMES (17)	3	9	4
DCX (354)	67	172	73	DCX (134)	26	64	38
NEUROD6 (273)	46	129	54	NEUROD6 (105)	23	52	32
STMN2 (312)	57	144	62	STMN2 (125)	25	61	34
RELN-L1 (28)	7	12	8	RELN-L1 (11)	1	5	3
CUX1-L2 (203)	37	101	47	CUX1-L2 (82)	21	42	27
POU3F2-L3 (BRN2) (92)	19	46	16	POU3F2 (BRN2)-L3 (84)	20	38	25
SATB2-L4 (45)	7	27	11	SATB2-L4 (113)	25	56	31
BCL11B-L5 (243)	38	112	52	BCL11B-L5 (71)	14	35	23
TLE4-L6 (120)	28	66	31	TLE4-L6 (72)	16	39	27
S100B (4)	0	3	5	S100B (6)	0	5	2
OLIG2 (0)	0	0	0	OLIG2 (2)	0	0	1
GFAP (7)	7	4	0	GFAP (19)	5	10	8

*(A) Table showing number of cells expressing marker(s) of interest at 12–13wpc and 16–18wpc. The values in the table represent the number of cells co-expressing markers of interest. L1–L6 are the cortical layers. Numbers in parenthesis represent the total number of cells for each marker*.

## Discussion

**Figure 4:**
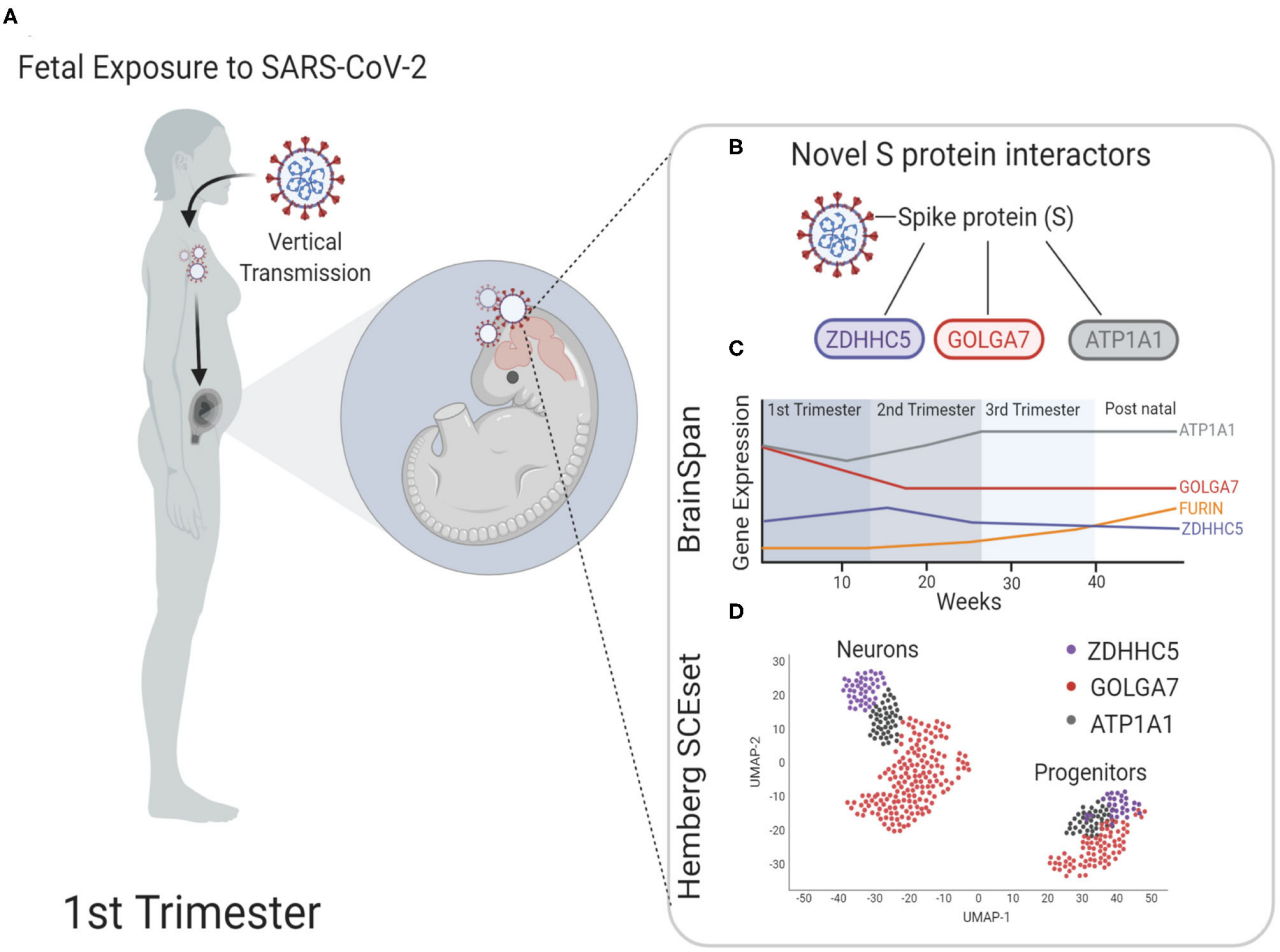
Model for possible infection of SARS-CoV-2 in fetal brain. **(A)** During the first trimester, pregnant mothers infected with SARS-CoV-2 can pass the virus through the placenta to the fetus via vertical transmission. It is unclear the health complications on the developing fetus this may cause or if the fetal brain is susceptible to SARS-CoV-2 neuroinvasion. **(B)** Schematic shows spike protein (S) of SARS-CoV-2 and the novel interacting proteins. **(C)** Time series expression data of novel S protein interactors to show the relative expression level analyzed from BrainSpan: Atlas of the Developing Human Brain database (https://brainspan.org/). Known interactors ACE2 and TMPRSS2 are not expressed in fetal brain tissue, but novel S protein interactors express mRNA transcripts at higher levels throughout fetal development. **(D)** Schematic of scRNA sequencing representing novel interactors identified in neurons and progenitors of fetal brain tissue analyzed from the fetal brain scRNA seq datasets maintained by Hemberg lab at Wellcome Trust Sanger Institute (https://hemberg-lab.github.io/scRNA.seq.datasets/human/brain/).

Pregnant women all over the world are at greater risk to COVID-19 infections due to altered immunity as well as strained pulmonary and cardiovascular system (Robinson and Klein, 2012). A recent study analyzed vertical transmission of SARS-CoV-2 during pregnancy in 31 subjects and found that although the rate of transmission was low, it was still possible and was accompanied by a strong inflammatory response (Kotlyar et al., 2020). Another study screened 101 women and found one positive case again highlighting the low but possible vertical transmission (Facchetti et al., 2020). Additionally, a case study published recently demonstrated that not only can fetuses be infected by SARS-CoV-2, the neonate born to infected mother can exhibit transient neurological complications (Vivanti et al., 2020). As more data is expected over time for short and long-term neurological complications in babies born to infected pregnant mothers during this pandemic, it is critical to understand the cellular and molecular mechanisms by which COVID-19 infection could potentially impact fetal brain development (Figure 4A). Using publicly available bulk and scRNA seq fetal brain transcriptome datasets, we showed that known S protein interactors *ACE2* and *TMPRSS2*, which play a key role in human pathogenesis, are expressed at low levels during fetal brain development. However, newly identified S protein interactors *ZDHHC5, GOLGA7*, and *ATP1A1* are abundantly expressed throughout fetal brain development with peaks of expression around 2^nd^ and 3^rd^ trimester of pregnancy (summarized in Figures 4B,C). Furthermore, we show that these S protein interactors are expressed both in neurons and progenitors with higher co-expression in neurons (summarized in Figure 4D). These results suggest that (1) *ZDHHC5, GOLGA7*, and *ATP1A1* could play a role in potential fetal brain SARS-CoV-2 pathogenesis, (2) 2^nd^ and 3^rd^ trimester of pregnancy could be more vulnerable for fetal brain infections and downstream targets of *ZDHHC5, GOLGA7*, and *ATP1A1* could lead to disruption of neuronal function.

SARS-CoV-2 employs the S protein, a heavily glycosylated type I membrane protein, for fusion into the host cells (Hoffmann et al., 2020). Furin-like proteases are believed to cleave S proteins into two fragments: the receptor-binding fragment S1 and the fusion fragment S2. Serine proteases such as TMPRSS2 or endosomal cysteine proteases are thought to trigger dissociation of S1 and bring a conformational change that facilitates the fusion of viral membrane and host cell membrane (Cai et al., 2020; Hoffmann et al., 2020). Using X-ray crystallography and cryo-EM imaging, recent studies have identified nine cysteine residues within the RBD, out of which eight form disulfide bonds (Lan et al., 2020). These cysteine residues in SARS-CoV have been shown to be important for viral invasion and a cysteine mutation to alanine disrupts fusion of viral membrane and host cell membrane. In addition, it has been shown that palmitoylation of cysteine residues is crucial for its fusogenic properties along with influences on localization, accumulation, secretion, stability, and membrane affinity of proteins (Petit et al., 2007). Modification to cysteine residues of S protein appear to be key regulators of SARS-CoV-2 and target cell interaction.

The newly identified S protein interactors ZDHHC5 and GOLGA7 form a protein acyltransferase complex which catalyzes protein-S-acylation of internal cysteine residues in various proteins (Swarthout et al., 2005; Kokkola et al., 2011). A study by Sergeeva et al., showed that ZDHHC5 activity is required for invasion of two very different toxins in host cells: the anthrax lethal toxin and the pore-forming toxin aerolysin. In both cases, ZDHHC5 functions by palmitoylating Furin that affects both the endocytic/recycling pathway and its association with plasma membrane microdomains (Sergeeva and van der Goot, 2019). As SARS-CoV-2 also requires Furin for S protein cleavage for viral entry into the host cell, the ZDHHC5-GOLGA7 complex could potentially play a role in palmitoylation of Furin and its recruitment to the plasma membrane. ZDHHC5 is also necessary for proper recruitment of nucleotide oligomerization domain (NOD)-like receptors 1 and 2 (NOD1/2) to the bacterial entry sites, key regulator of innate immune response to pathogen entry (Lu et al., 2019). Moreover, ZDHHC5 has been shown to localize to synaptic vesicles and interact with PSD-95, a postsynaptic membrane protein (Li et al., 2010; Brigidi et al., 2014, 2015). It has been shown to specifically palmitoylate *GRIP1b* to accelerate postsynaptic AMPA receptor recycling (Thomas et al., 2012), a neurological process implicated in the mechanism for autism (Mejias et al., 2011). Further, mice with a hypomorphic allele of *ZDHHC5* showed impaired learning and memory (Li et al., 2010).

The association of ZDHHC5 and GOLGA7 with the S protein of SARS-CoV-2 suggests two possibilities. They could act directly, where in the absence of known S protein interactors (ACE2 and TMPRSS2), other membrane-associated proteins such as ZDHHC5 and GOLGA7 could function as receptors for invasion into neuronal or neural progenitor cells. This process could be facilitated by palmitoylation of S protein cysteine residues and/or Furin. Alternately, it could act indirectly, where sequestration of ZDHHC5 and GOLGA7 by S protein of SARS-CoV-2 could compromise the physiological functions of these proteins, leading to short and long-term impact of SARS-CoV-2 infection in fetal brain. For instance, interaction of ZDHHC5-GOLGA7 complex with S protein can disrupt the stochiometric interaction of ZDHHC5 with PSD-95 impacting its downstream physiological processes resulting in impaired learning and memory. Further cellular and molecular analysis of these genes will be required to test these possibilities.

ATP1A1, the third novel S protein interactor, is a well-studied Na^+^/K^+^ ion transporter and is also involved in signal transduction pathways (Kaplan, 2002). It has been shown that blocking ATP1A1 by gene silencing or chemical compounds can inhibit CoV infection at early stages (Burkard et al., 2015). ATP1A1 is also essential for entry of respiratory syncytial virus (RSV) in human respiratory epithelial cells, where ATP1A1 transactivates downstream signaling pathways, resulting in the host cell taking up RSV particles through macropinocytosis (Lingemann et al., 2019). We found that ATP1A1 is expressed throughout fetal brain development with high expression at 21, 24, and 37wpc, suggesting that ATP1A1 could be an access point for SARS-CoV-2 infection of fetal brain.

Cerebral organoids mimic transcriptional and proteomic signatures of fetal brain development and have grown in popularity as excellent models to understand neurodevelopmental disorders in humans (Camp et al., 2015; Kelava and Lancaster, 2016; Luo et al., 2016; Kanton et al., 2019; Nascimento et al., 2019; Logan et al., 2020). Recently cerebral organoids were infected with SARS-CoV-2 providing the first evidence of neurotropism in *in vitro* cultures (Jacob et al., 2020; Pellegrini et al., 2020; Ramani et al., 2020; Song et al., 2020; Zhang et al., 2020). However, cerebral organoids express extremely low levels of *ACE2*, which leaves open the question of how SARS-CoV-2 invades neurons (Ramani et al., 2020). The newly identified S protein interactors ZDHHC5, GOLGA7, and ATP1A1 are strong candidates for further analysis in this system, which could provide more insight into fetal neuroinvasion by SARS-CoV-2. Co-immunoprecipitation studies in SARS-CoV-2 infected cerebral organoids can confirm the interaction of these novel proteins with S protein. Additionally, knockdown of these interactors in cerebral organoids can help identify their role in SARS-CoV-2 fetal brain pathogenesis.

To conclude, we have analyzed the expression of known and novel S protein interactors of SARS-CoV-2 in fetal brain development. Though two of the known S protein interactors are nominally expressed during fetal brain development, there is abundant expression of three newly identified interactors. As these interactors play crucial physiological roles in human brain, their interaction with SARS-CoV-2 can have critical downstream consequences during fetal neurodevelopment. Moreover, as these genes are most abundantly expressed between 12 and 26wpc, our study suggests that fetal brain during 2^nd^ and 3^rd^ trimester of pregnancy could be more vulnerable to COVID-19 infection. Further detailed cellular and molecular analysis of these genes will show whether they are necessary and sufficient for SARS-CoV-2 infection in fetal brain.

## Data Availability Statement

Publicly available datasets were analyzed in this study. This data can be found at: (1) BrainSpan: Atlas of the Developing Human Brain (https://brainspan.org/) (2) https://hemberg-lab.github.io/scRNA.seq.datasets/human/brain/.

## Ethics Statement

This study has performed only secondary data analysis. However, the original articles that published the primary datasets reviewed and approved the protocols related to postmortem human brain specimens. Written informed consent was not required for this study in accordance with the national legislation and institutional requirements.

## Author Contributions

PV, ZL, and JH designed research and wrote the paper. PV and MA performed research and data analysis. All authors contributed to the article and approved the submitted version.

## Conflict of Interest

The authors declare that the research was conducted in the absence of any commercial or financial relationships that could be construed as a potential conflict of interest.

## Supplementary Material

Supplementary Figure 1 **(A)** UMAP of 12–13wpc dataset with expression levels of known S protein interactors *ACE2, TMPRSS2*, and *FURIN*. ACE2 and TMPRSS2 are not expressed at 12–13wpc. **(B**) UMAP of 16–18wpc dataset with expression levels of known S protein interactors *ACE2, TMPRSS2*, and *FURIN*. ACE2 *and* TMPRSS2 are not expressed at 16–18wpc.
