# Supplementary figures and images for: Novel Targets of SARS-CoV-2 Spike Protein in Human Fetal Brain Development Suggest Early Pregnancy Vulnerability
Source: Front Neurosci. 2021 Jan 21;14:614680. doi: 10.3389/fnins.2020.614680 (PMC7859280; doi:10.3389/fnins.2020.614680)

Supplementary Figure 1

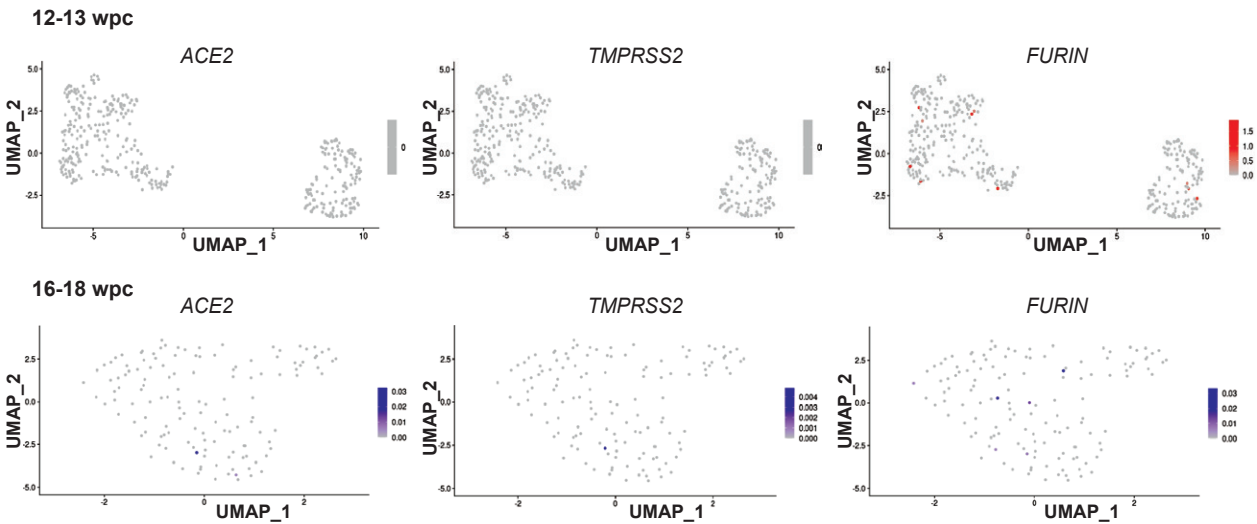

Supplement: Supplementary Figure 1 — (A) UMAP of 12–13wpc dataset with expression levels of known S protein interactors ACE2, TMPRSS2, and FURIN. ACE2 and TMPRSS2 are not expressed at 12–13wpc. (B) UMAP of 16–18wpc dataset with expression levels of known S protein interactors ACE2, TMPRSS2, and FURIN. ACE2 and TMPRSS2 are not expressed at 16–18wpc. [file Data_Sheet_1.PDF]
